# Blood pressure and mortality in patients with type 2 diabetes and a recent coronary event in the ELIXA trial

**DOI:** 10.1186/s12933-020-01150-0

**Published:** 2020-10-12

**Authors:** Magnus O. Wijkman, Brian Claggett, Rafael Diaz, Hertzel C. Gerstein, Lars Køber, Eldrin Lewis, Aldo P. Maggioni, Emil Wolsk, David Aguilar, Rhonda Bentley-Lewis, John J. McMurray, Jeffrey Probstfield, Matthew Riddle, Jean-Claude Tardif, Scott D. Solomon, Marc A. Pfeffer

**Affiliations:** 1 Cardiovascular Division, Brigham & Women’s Hospital, Harvard Medical School, 75 Francis Street, Boston, MA 02115 USA; 2 Department of Internal Medicine and Department of Health, Medicine and Caring Sciences, Linköping University, Norrköping, Sweden; 3 Estudios Clínicos Latinoamérica, Instituto Cardiovascular de Rosario, Rosario, Argentina; 4 The Population Health Research Institute and the Department of Medicine, McMaster University and Hamilton Health Sciences, Hamilton, Canada; 5 Rigshospitalet Copenhagen University Hospital, Copenhagen, Denmark; 6 Stanford University Medical Center, Stanford, USA; 7 Maria Cecilia Hospital, GVM Care & Research, Cotignola, Italy; 8 Research Center of the Italian Association of Hospital Cardiologists, Florence, Italy; 9 McGovern Medical School, University of Texas, Houston, USA; 10 Massachusetts General Hospital, Harvard Medical School, Boston, USA; 11 British Heart Foundation Cardiovascular Research Centre, University of Glasgow, Glasgow, UK; 12 University of Washington Medical Center, Seattle, USA; 13 Oregon Health and Science University, Portland, USA; 14 Montreal Heart Institute, Université de Montréal, Montreal, Canada

**Keywords:** Diabetes mellitus, Coronary artery disease, Blood pressure

## Abstract

**Background:**

The relationship between blood pressure and mortality in type 2 diabetes (T2DM) is controversial, with concern for increased risk associated with excessively lowered blood pressure.

**Methods:**

We evaluated whether prior cardiovascular disease (CVD) altered the relationship between baseline blood pressure and all-cause mortality in 5852 patients with T2DM and a recent acute coronary syndrome (ACS) who participated in the ELIXA (Evaluation of Lixisenatide in Acute Coronary Syndrome) trial. Risk of death was assessed in Cox models adjusted for age, sex, race, heart rate, BMI, smoking, diabetes duration, insulin use, HbA1c, eGFR, brain natriuretic peptide (BNP), urine albumin/creatinine ratio, treatment allocation and prior coronary revascularization.

**Results:**

Although overall there was no significant association between systolic blood pressure (SBP) and mortality (hazard ratio per 10 mmHg lower SBP 1.05 (95% CI 0.99–1.12) P = 0.10), lower SBP was significantly associated with higher risk of death (hazard ratio per 10 mmHg lower SBP 1.13 (95% CI 1.04–1.22) P = 0.002) in 2325 patients with additional CVD (index ACS+ at least one of the following prior to randomization: myocardial infarction other than the index ACS, stroke or heart failure). In 3527 patients with only the index ACS no significant association was observed (hazard ratio per 10 mmHg lower SBP 0.95 (0.86–1.04) P = 0.26; P for interaction 0.005).

**Conclusions:**

The association between blood pressure and mortality was modified by additional CVD history in patients with type 2 diabetes and a recent coronary event. When blood pressures measured after an acute coronary event are used to assess the risk of death in patients with type 2 diabetes, the cardiovascular history needs to be taken into consideration.

*Trial registration* ClinicalTrials.gov number NCT01147250, first posted June 22, 2010

## Background

Elevated blood pressure is considered an important additive risk factor in patients with type 2 diabetes, augmenting the already heightened risk for morbidity and mortality in these patients [1]. However, there is ongoing controversy regarding the optimal blood pressure treatment targets [2], and current guidelines provide differing recommendations [3–5]. In patients with type 1 diabetes, a linear relationship between higher systolic blood pressure and higher risk for stroke has been observed even below blood pressure levels recommended in more strict guidelines [6]. Observational data have shown linear relationships between higher blood pressure and higher cardiovascular risk in patients with type 2 diabetes without a history of cardiovascular disease (CVD) [7], whereas U-shaped relationships have been described in patients with type 2 diabetes and established CVD or in patients with a high-risk profile [8–12]. Similar concerns for higher cardiovascular risk with excessively lowered systolic or diastolic blood pressures have been raised for patients with established coronary artery disease [13, 14]. However, little is known about the associations between blood pressure measured after an acute coronary event and subsequent risk of death in patients with type 2 diabetes, and whether a history of additional CVD may have an impact on the association between blood pressure levels and mortality in these patients. Therefore, the aims of this study were twofold: to characterize the overall relationships between baseline blood pressure levels and all-cause mortality in patients with type 2 diabetes who, as required for entry to the ELIXA (Evaluation of Lixisenatide in Acute Coronary Syndrome) trial, had a recent acute coronary syndrome (ACS), and to ascertain whether these relationships were influenced by prior CVD status.

## Methods

### Patients

The design, patient characteristics and cardiovascular outcomes of the ELIXA trial (ClinicalTrials.gov number NCT01147250) have been published previously [15, 16]. In brief, ELIXA was a randomized placebo-controlled double-blind multi-center trial which evaluated the cardiovascular safety and outcomes of treatment with lixisenatide, a glucagon-like peptide 1-receptor agonist in 6068 patients with type 2 diabetes who had recently experienced an ACS. Exclusion criteria were age of less than 30 years, glycated hemoglobin level of less than 5.5% or more than 11.0%, estimated glomerular filtration rate (eGFR) of less than 30 ml/min/1.73 m^2^, coronary artery by-pass graft surgery for the qualifying event, percutaneous coronary intervention within 15 days prior to the screening visit, planned coronary revascularization within 90 days after the screening visit, or inability to provide written informed consent. There were no exclusion criteria related to blood pressure levels. The median follow-up time was 25 months. Lixisenatide (or volume-matched placebo) was administered as sub-cutaneous injections, with a maximal daily dose of 20 µg/day. For the purpose of this secondary analysis, 216 patients were excluded due to missing baseline data (reasons for exclusion are presented in Additional file 1: Table S1) and this yielded a study cohort of 5852 patients. These patients were divided in two groups: those who prior to randomization had a history of at least one of the following: myocardial infarction other than the index ACS, stroke or heart failure (“additional CVD”), and those who did not have a history of any of the above prior to randomization (“index ACS only”).

### Measurements of blood pressure and left ventricular ejection fraction

Baseline blood pressure was assessed as the mean of screening and randomization blood pressures. The median number of days between the screening and randomization visits was seven (interquartile range [IQR] 7–8 days). The study instructions stated that blood pressure measurements should be performed with the patient in the supine position, after the patient had rested comfortably for at least 5 min. Pulse pressure (PP) was calculated as systolic blood pressure (SBP) minus diastolic blood pressure (DBP). Throughout the trial, the antihypertensive medications could be adjusted according to local practice guidelines, as deemed appropriate by the patient´s treating physicians. When available, left ventricular ejection fraction (LVEF) was reported by the enrolling sites on an electronic case report form, as described previously [17].

### Statistics

Descriptive data are presented as mean ± standard deviation (SD) and/or as median [IQR] for continuous variables. Between-group differences were tested for statistical significance with Student’s t-test, Wilcoxon rank sum test, analysis of variance or Kruskal–Wallis test for continuous variables, and with Chi square test for categorical variables. The incidence of death was plotted against SBP, DBP, and PP, using restricted cubic spline curves with three knots. The associations were tested for overall statistical significance and for evidence of statistically significant non-linearity based on the Poisson distributions of the incidence rates. A proportional hazards Cox regression model was used to calculate the hazard ratio (HR) for death with 95% confidence intervals (CI) associated with a blood pressure difference of 10 mmHg. For significant associations that also exhibited evidence of significant non-linearity, piecewise Cox regression was used to determine hazard ratios separately for the lower and the upper parts of the spline curves. All spline curves and Cox regressions were adjusted for randomization group and for history of coronary revascularization prior to randomization and for the following potential baseline confounders, that were selected from a previously published risk prediction model [18]: age, sex, self-reported race (Asian/Black or African American/White/Other), heart rate, body mass index (BMI), smoking status (Never/Former/Current), duration of diabetes, glycated hemoglobin A1c, use of insulin, estimated glomerular filtration rate (eGFR), the logarithm of the urinary albumin to creatinine ratio and the logarithm of the brain-type natriuretic peptide (BNP) level. By study design, all patients in ELIXA were included after hospitalization for an ACS. In this analysis, we first analyzed the relationships between blood pressure and mortality in all patients, and then repeated the analyses separately in patients without a history of additional CVD prior to randomization (“index ACS only”) and in patients who prior to randomization did have a history of at least one of the following: myocardial infarction other than the index ACS, stroke, or heart failure (“additional CVD”). Interactions between blood pressure and additional CVD history were tested for statistical significance with stratified Cox regression. A sensitivity analysis was also performed in patients who did not have heart failure at baseline, and another sensitivity analysis in which we additionally adjusted for LVEF was performed in the 3957 patients where this information was available. When testing for statistical significance, we considered P values < 0.05 as significant. No correction was made for multiple testing. Statistical analyses were performed in STATA, version 14 (College Station, TX).

## Results

### Patient characteristics

**Fig. 1:**
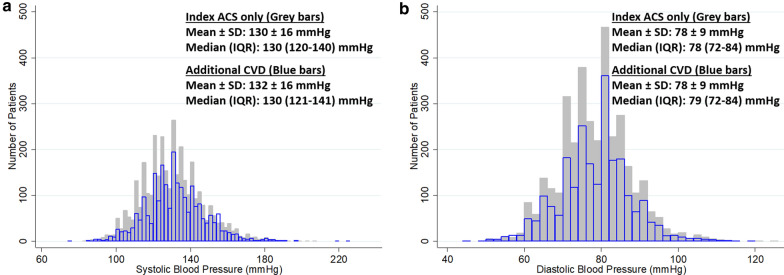
Baseline distributions of systolic (**a**) and diastolic (**b**) blood pressures. Grey bars show patients with index acute coronary syndrome (ACS) only (n = 3527). Blue bars show patients with additional cardiovascular disease (CVD) (n = 2325)

Overall, the mean age was 60.3 ± 9.7 years and 1779 patients (30.4%) were women. The median number of days between the index ACS and the first blood pressure measurement was 52 [IQR: 30–86 days]. Baseline SBP was less than 140 mmHg in 4256 patients (72.7%) and baseline DBP was less than 90 mmHg in 5273 patients (90.1%). There were 2325 patients (39.7%) who prior to randomization had a history of additional CVD (either myocardial infarction other than the index ACS (n = 1341) and/or stroke (n = 314) and/or heart failure (n = 1317)). Baseline patient characteristics are presented in Table 1 according to prior CVD status, and in Table 2 according to tertiles of SBP. Despite having lower SBP and lower DBP, patients in the lowest tertile of SBP were less likely to use calcium channel blockers, angiotensin receptor blockers and diuretics (Table 2). The baseline distributions of SBP and DBP are shown graphically in Fig. 1 according to prior CVD status. At baseline, the mean SBP was 130 ± 16 [median 130, IQR: 120–140] mmHg in patients with index ACS only and 132 ± 16 [median 130, IQR: 121–141] mmHg in patients with additional CVD, P < 0.001. The baseline mean DBP was 78 ± 9 [median 78, IQR: 72–84] mmHg in patients with index ACS only and 78 ± 9 [median 79, IQR: 72–84] mmHg in patients with additional CVD, P = 0.95.

**Table 1:** Baseline characteristics by prior additional CVD status

	Index ACS only n = 3527	Additional CVD n = 2325	P
Age (years)	59.0 ± 9.6	62.3 ± 9.3	< 0.001
Female, n (%)	980 (28%)	799 (34%)	< 0.001
Race, n (%)			< 0.001
Asian	582 (17%)	176 (8%)	
Black or African American	116 (3%)	88 (4%)	
White	2501 (71%)	1907 (82%)	
Other	328 (9%)	154 (7%)	
Systolic blood pressure (mmHg)	130 ± 16	132 ± 16	< 0.001
Diastolic blood pressure (mmHg)	78 ± 9	78 ± 9	0.95
Pulse pressure (mmHg)	52 ± 13	54 ± 13	< 0.001
Heart rate (bpm)	70 ± 9	70 ± 9	0.89
BMI (kg/m^2^)	29.7 ± 5.7	30.7 ± 5.6	< 0.001
Diabetes duration (years)	8.5 ± 7.8	10.4 ± 8.7	< 0.001
HbA1c (%)	7.7 ± 1.3	7.7 ± 1.3	0.48
eGFR (ml/min/1.73m^2^)	79 ± 21	71 ± 21	< 0.001
Albuminuria (mg/g)			< 0.001
< 30 (normal)	2697 (77%)	1633 (70%)	
≥ 30–< 300 (micro)	631 (18%)	494 (21%)	
≥ 300 (macro)	184 (5%)	191 (8%)	
BNP (pg/ml) median [IQR]	90 [44–176]	138 [63–293]	< 0.001
Smoking status, n (%)			0.005
Current	421 (12%)	246 (11%)	
Former	1646 (47%)	1019 (44%)	
Never	1460 (41%)	1060 (46%)	
MI history other than index ACS, n (%)	0 (0.0%)	1341 (58%)	NA
Stroke history, n (%)	0 (0.0%)	314 (14%)	NA
Heart failure history, n (%)	0 (0.0%)	1317 (57%)	NA
Coronary revascularization history, n (%)	2527 (72%)	1554 (67%)	< 0.001
STEMI at index event, n (%)	1817 (52%)	756 (33%)	< 0.001
Left ventricular ejection fraction^a^ (percent)	53 ± 11	48 ± 13	< 0.001
Insulin, n (%)	1239 (35%)	1042 (45%)	< 0.001
Statin, n (%)	3333 (95%)	2099 (90%)	< 0.001
Beta blocker, n (%)	2904 (82%)	2049 (88%)	< 0.001
Calcium channel blocker, n (%)	652 (19%)	629 (27%)	< 0.001
Diuretic, n (%)	1085 (31%)	1223 (53%)	< 0.001
ACE inhibitor, n (%)	2095 (59%)	1437 (62%)	0.07
ARB, n (%)	910 (26%)	645 (28%)	0.10
Alpha blocker, n (%)	136 (4%)	122 (5%)	0.011
Potassium sparing agents, n (%)	347 (10%)	508 (22%)	< 0.001

Unless stated otherwise, values are mean ± SD or n (%)

*ACE* angiotensin converting enzyme, *ACS* acute coronary syndrome, *ARB* angiotensin receptor blocker, *BMI* body mass index, *BNP* brain-type natriuretic peptide, *eGFR* estimated glomerular filtration rate, *HbA1c* glycosylated hemoglobin, *HDL* high density lipoprotein, *LDL* low density lipoprotein, *MI* myocardial infarction, *STEMI* ST segment elevation myocardial infarction

^a^Data available for 3957 patients for left ventricular ejection fraction (2352 in patients with index ACS only and 1605 in patients with additional CVD)

**Table 2:** Baseline characteristics by tertiles of systolic blood pressure

	Tertile 1 n = 2008 SBP 74–124 mmHg	Tertile 2 n = 1909 SBP > 124–136 mmHg	Tertile 3 n = 1935 SBP > 136–225 mmHg	P
Age (years)	57.9 ± 9.8	60.5 ± 9.3	62.6 ± 9.3	< 0.001
Female, n (%)	503 (25%)	603 (32%)	673 (35%)	< 0.001
Race, n (%)				< 0.001
Asian	294 (15%)	274 (14%)	190 (10%)	
Black or African American	52 (3%)	74 (4%)	78 (4%)	
White	1444 (72%)	1447 (76%)	1517 (78%)	
Other	218 (11%)	114 (6%)	150 (8%)	
Systolic blood pressure (mmHg)	114 ± 8	130 ± 4	149 ± 11	NA
Diastolic blood pressure (mmHg)	72 ± 7	79 ± 7	84 ± 9	< 0.001
Pulse pressure (mmHg)	42 ± 7	51 ± 7	65 ± 12	NA
Heart rate (bpm)	71 ± 9	70 ± 9	70 ± 10	0.011
BMI (kg/m^2^)	29.1 ± 5.3	30.4 ± 5.8	31.0 ± 5.8	< 0.001
Diabetes duration (years)	8.4 ± 8.0	9.2 ± 8.1	10.3 ± 9	< 0.001
HbA1c (%)	7.7 ± 1.3	7.7 ± 1.3	7.7 ± 1.3	0.33
eGFR (ml/min/1.73m^2^)	78 ± 21	76 ± 21	74 ± 22	< 0.001
Albuminuria (mg/g)				< 0.001
< 30 (normal)	1637 (82%)	1424 (75%)	1269 (66%)	
≥ 30–< 300 (micro)	297 (15%)	363 (19%)	465 (24%)	
≥ 300 (macro)	67 (3%)	117 (6%)	191 (10%)	
BNP (pg/ml) median [IQR]	106 [49–234]	100 [47–203]	111 [54–226]	0.006
Smoking status, n (%)				< 0.001
Current	248 (12%)	214 (11%)	205 (11%)	
Former	1012 (50%)	789 (41%)	864 (45%)	
Never	748 (37%)	906 (48%)	866 (45%)	
MI history other than index ACS, n (%)	402 (20%)	450 (24%)	489 (25%)	< 0.001
Stroke history, n (%)	73 (4%)	106 (6%)	135 (7%)	< 0.001
Heart failure history, n (%)	436 (22%)	469 (25%)	412 (21%)	0.030
Coronary revascularization history, n (%)	1449 (72%)	1241 (65%)	1391 (72%)	< 0.001
STEMI at index event, n (%)	1088 (54%)	798 (42%)	687 (36%)	< 0.001
Left ventricular ejection fraction^a^ (percent)	48 ± 13	52 ± 12	52 ± 11	< 0.001
Insulin, n (%)	733 (37%)	729 (38%)	819 (42%)	< 0.001
Statin, n (%)	1889 (94%)	1755 (92%)	1788 (92%)	0.024
Beta blocker, n (%)	1691 (84%)	1597 (84%)	1665 (86%)	0.10
Calcium channel blocker, n (%)	224 (11%)	428 (22%)	629 (33%)	< 0.001
Diuretic, n (%)	727 (36%)	728 (38%)	853 (44%)	< 0.001
ACE inhibitor, n (%)	1233 (61%)	1164 (61%)	1135 (59%)	0.17
ARB, n (%)	418 (21%)	503 (26%)	634 (33%)	< 0.001
Alpha blocker, n (%)	89 (4%)	67 (4%)	102 (5%)	0.029
Potassium sparing agents, n (%)	367 (18%)	268 (14%)	220 (11%)	< 0.001

Unless stated otherwise, values are mean ± SD or n (%)

*ACE* angiotensin converting enzyme, *ACS* acute coronary syndrome, *ARB* angiotensin receptor blocker, *BMI* body mass index, *BNP* brain-type natriuretic peptide, *eGFR* estimated glomerular filtration rate, *HbA1c* glycosylated hemoglobin, *HDL* high density lipoprotein, *LDL* low density lipoprotein, *MI* myocardial infarction, *SBP* systolic blood pressure, *STEMI* ST segment elevation myocardial infarction

^a^Data available for 3957 patients for left ventricular ejection fraction (1424 in tertile 1, 1262 in tertile 2 and 1271 in tertile 3)

### Blood pressure and mortality

Overall, there were 421 deaths among the 5852 patients in this analysis (3.3 deaths per 100 patient-years). There were more deaths among the 2325 patients with additional CVD (258 deaths, 5.0 deaths per 100 patient-years) than among the 3527 patients with index ACS only (163 deaths, 2.1 deaths per 100 patient-years) P < 0.001. The number of deaths did not differ significantly between tertiles of SBP (first tertile: 136 deaths/2008 patients, 3.1 deaths per 100 patient-years; second tertile: 141 deaths/1909 patients, 3.3 deaths/100 patient-years; third tertile: 144 deaths/1935 patients, 3.4 deaths/100 patient-years) P = 0.66.

**Fig. 2:**
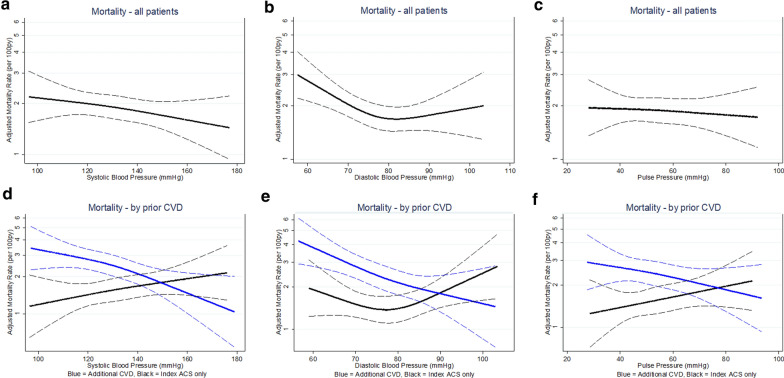
Adjusted mortality rates and baseline blood pressures. Upper row: entire cohort, **a** systolic blood pressure, **b** diastolic blood pressure, **c** pulse pressure. Lower row: stratified by prior cardiovascular disease (CVD), **d** systolic blood pressure, **e** diastolic blood pressure, **f**  pulse pressure. Blue = Additional CVD, black = Index ACS only. P values for interaction: 0.005 (SBP), 0.004 (DBP), 0.12 (PP). Adjustments made for: randomization group, coronary revascularization history, age, sex, self-reported race, heart rate, BMI, smoking status, known duration of diabetes, glycated hemoglobin A1c, use of insulin, eGFR, the logarithm of the urinary albumin to creatinine ratio and the logarithm of the BNP level

Out of the 421 deaths, 307 were cardiovascular deaths (2.4 cardiovascular deaths per 100 patient-years). Overall, there was no significant association between SBP and mortality (Fig. 2, Table 3). However, the relationship between SBP and mortality differed according to prior CVD status (P for interaction 0.005) such that in patients with additional CVD, lower SBP was associated with higher mortality (HR per 10 mmHg lower SBP: 1.13 (1.04–1.22) P = 0.002) whereas in patients with index ACS only there was no significant relationship between SBP and mortality (Fig. 2, Table 3). For DBP, overall, there was a significant, non-linear, relationship between DBP and mortality such that for DBP values lower than 80 mmHg, lower DBP was associated with higher mortality (HR per 10 mmHg lower DBP: 1.30 (1.11–1.51) P = 0.001), but for DBP values of 80 mmHg and higher, no significant relationship was observed (Fig. 2, Table 3). The association between DBP and mortality also differed according to prior CVD status (P for interaction 0.004). In patients with additional CVD, the association changed from non-linear to linear, such that lower DBP was associated with higher mortality across the entire range of DBP values (HR per 10 mmHg lower DBP: 1.30 (1.13–1.49) P < 0.001) whereas in patients with index ACS only, there was no significant association between DBP and mortality (Fig. 2, Table 3). For PP, there was no significant association with mortality and no significant interaction between prior CVD and PP (Fig. 2, Table 3).

**Table 3:** Adjusted hazard ratios associated with 10 mmHg lower blood pressure

	Overall (n = 5852) HR (95% CI) P Per 10 mmHg ↓	Index ACS only (n = 3527) HR (95% CI) P Per 10 mmHg ↓	Additional CVD (n = 2325) HR (95% CI) P Per 10 mmHg ↓	P for interaction
Systolic blood pressure	1.05 (0.99–1.12) P = 0.10	0.95 (0.86–1.04) P = 0.26	1.13 (1.04–1.22) P = 0.002	0.005
Diastolic blood pressure	*	0.94 (0.79–1.12) P = 0.48	1.30 (1.13–1.49) P < 0.001	0.004
For DBP < 80 mmHg	1.30 (1.11–1.51) P = 0.001	–	–	
For DBP ≥ 80 mmHg	0.91 (0.74–1.13) P = 0.39	–	–	
Pulse pressure	1.02 (0.94–1.10) P = 0.65	0.95 (0.84–1.07) P = 0.36	1.07 (0.97–1.18) P = 0.19	0.12

^*^Denotes significantly non-linear associations, where two separate hazard ratios (obtained by piece-wise Cox regression) are reported. Adjustments made for: randomization group, coronary revascularization history, age, sex, self-reported race, heart rate, BMI, smoking status, known duration of diabetes, glycated hemoglobin A1c, use of insulin, eGFR, the logarithm of the urinary albumin to creatinine ratio and the logarithm of the BNP level

### Sensitivity analysis in patients without heart failure

After excluding 1317 patients with a diagnosis of heart failure at baseline, we performed a sensitivity analysis in the remaining 4535 patients (3527 with index ACS only and 1008 with additional CVD). There were 77 deaths in the group with additional CVD (3.5 deaths per 100 patient-years) and 163 deaths in the group with index ACS only (2.1 deaths per 100 patient-years) P < 0.001. As in the original cohort, there was no significant overall association between SBP and mortality (Additional file 1: Table S2) and there was a significant interaction (P < 0.001) between SBP and prior CVD. In patients with additional CVD, lower SBP remained significantly associated with higher mortality (HR per 10 mmHg lower SBP 1.39 (1.18–1.63) P < 0.001). For DBP, the overall relationship with mortality remained non-linear as in the original cohort (Additional file 1: Table S2) and the association between DBP and mortality also differed according to prior CVD status (P for interaction < 0.001). The relationship between lower DBP and higher mortality was linear in patients with additional CVD (HR per 10 mmHg lower DBP 1.82 (1.40–2.36) P < 0.001). For PP, there was no overall significant association with mortality (Additional file 1: Table S2), but there was a significant interaction (P = 0.036) between PP and prior CVD.

### Sensitivity analysis in patients with known left ventricular ejection fraction

In this study cohort, there were 3957 patients for whom LVEF after the index ACS was available (2352/3527 in patients with index ACS only (67%) and 1605/2325 in patients with additional CVD (69%), P = 0.06). The baseline distributions of LVEF are shown graphically in Additional file 1: Figure S1 according to prior CVD status. Mean LVEF was 53 ± 11 [median 53, IQR: 45–60] percent in patients with index ACS only and 48 ± 13 [median 50, IQR: 40–57] percent in patients with additional CVD, P < 0.001. There were 167 deaths in the group with additional CVD (4.7 deaths per 100 patient-years) and 115 deaths (2.2 deaths per 100 patient-years) in the group with index ACS only, P < 0.001. We performed a sensitivity analysis in these patients, in which we additionally adjusted for LVEF. Overall, lower systolic blood pressure was significantly associated with higher mortality (HR per 10 mmHg lower SBP: 1.09 (1.01–1.18) P = 0.028). The relationship between SBP and mortality differed according to prior CVD status (P for interaction 0.020) such that in patients with additional CVD, lower SBP was associated with higher mortality (HR per 10 mmHg lower SBP: 1.18 (1.07–1.32) P = 0.001), whereas in patients with index ACS only, there was no significant relationship between SBP and mortality (Additional file 1: Table S3). For DBP, overall, there was a significant linear relationship between lower DBP and higher mortality (HR per 10 mmHg lower DBP: 1.17 (1.02–1.34) P = 0.025). As in the original cohort, the association between DBP and mortality also differed according to prior CVD status (P for interaction 0.022). In patients with additional CVD, there was a significant linear association between lower DBP and higher mortality (HR per 10 mmHg lower DBP: 1.34 (1.12–1.60) P = 0.001) whereas in patients with index ACS only, there was no significant association between DBP and mortality (Additional file 1: Table S3). For PP, there was no overall significant association with mortality and no significant interaction between prior CVD and PP (Additional file 1: Table S3).

## Discussion

### Main findings

The main finding of this study was that in ELIXA participants with type 2 diabetes and a recent acute coronary event, there were no overall significant relationships between SBP or PP and mortality, but there was a non-linear relationship between DBP and mortality, such that lower DBP was associated with higher mortality for DBP levels lower than 80 mmHg and no significant relationship was observed for DBP values of 80 mmHg and higher. However, a greater burden of CVD, as defined by prior CVD in addition to the index event, modified the relationship between blood pressure and risk of death. Independently of several known predictors of death, lower baseline SBP or lower baseline DBP were significantly associated with higher mortality in patients with a history of additional CVD, but not in patients without additional prior CVD. A greater burden of CVD modified the relationship between blood pressure and risk of death also in a sensitivity analysis in which patients with heart failure were excluded, and in a sensitivity analysis of patients for whom additional adjustment for LVEF was made. Compared with patients with additional CVD, patients without additional CVD had lower systolic blood pressure and a more favorable risk factor profile: they were, on average, younger, had lower BMI, higher eGFR, shorter duration of diabetes, and were less often treated with insulin despite having similar HbA1c, and more often treated with a statin. These differences may have contributed to the lower event rates in the group without additional CVD and may explain why patients with additional CVD appeared to be more vulnerable to the detrimental effects of low blood pressure.

### Effects of lower blood pressure in coronary artery disease

Coronary perfusion occurs mainly during diastole, and lower diastolic blood pressure has been associated with higher odds for angina in patients with coronary artery disease [19]. Diabetes status has been shown to modify the relationship between diastolic blood pressure and collateral flow index in patients with chronic total occlusion, so that at various degrees of stenosis of the predominant collateral donor artery, the collateral flow index decreased more with decreasing diastolic blood pressure in patients with diabetes than in patients without diabetes [20]. This suggests that in patients with coronary artery disease, lower diastolic blood pressure may be even more important as a risk factor in patients who also have diabetes. In patients with stable coronary artery disease who participated in the INVEST (International Verapamil SR-Trandolapril Study) trial, there was a linear relationship between lower diastolic blood pressure and lower risk for cardiovascular events or death in patients who had undergone coronary artery by-pass graft surgery only, whereas in all other patients, the relationship was J-shaped, with increasing risk in patients with lower diastolic blood pressure [21]. In INVEST, the prevalence of angina was also lower in patients with a history of coronary artery by-pass graft surgery [21]. This may suggest that in patients with stable coronary artery disease, those who have undergone more complete revascularization may tolerate lower diastolic blood pressure better. This hypothesis is indirectly supported by the present study, in which lower diastolic blood pressure was significantly associated with higher mortality only in patients with additional CVD, a group of patients who were also less likely to have had prior coronary revascularizations.

### Randomized trials of blood pressure targets in diabetes

In patients with diabetes, randomized trials that compared different blood pressure treatment targets have not shown a clear benefit of lower treatment targets on survival [22–24]. Benefits on combined morbidity and mortality outcomes were observed in the UKPDS (United Kingdom Prospective Diabetes Study) trial where average baseline blood pressure was 160/94 mmHg [22], and in a subgroup of the HOT (Hypertension Optimal Treatment) trial where the baseline DBP inclusion criteria was 100–115 mmHg [23], but not in the more recent ACCORD (Action to Control Cardiovascular Risk in Diabetes) trial, where average baseline blood pressure was 139/76 mmHg [24] and in which a systolic treatment target of less than 120 mmHg did not influence the risk for major cardiovascular events compared with a treatment target of less than 140 mmHg. One meta-analysis showed that in patients with diabetes, initiation of antihypertensive treatment provided clinical benefit only in patients with pre-treatment SBP ≥ 140 mmHg, and that treatment intensification actually increased the risk for cardiovascular death in patients with SBP less than 140 mmHg [25]. The results of the present study are observational and should not be interpreted as evidence for or against intensified blood pressure treatment in patients with diabetes. However, the results do suggest that an association between lower SBP and higher risk for death may be confined to patients with more advanced cardiovascular disease burden, and that elevated blood pressure may be a more important risk factor in patients without prior cardiovascular events. Importantly, our results should not discourage the use of blood pressure-lowering medications known to prolong survival. For instance, in patients with heart failure who participated in the CHARM (Candesartan in Heart Failure Assessment of Reduction in Mortality and Morbidity) trials, lower baseline blood pressure was also associated with higher risk for death, but the benefits of treatment with candesartan were not affected by baseline blood pressure levels [26].

### Observational studies of blood pressure levels and risk in diabetes

In a secondary analysis of high risk patients with both type 2 diabetes, hypertension and coronary artery disease who participated in the HIJ-CREATE (The Heart Institute of Japan Candesartan Randomized Trial for Evaluation in Coronary Artery Disease) trial, achievement of strict systolic blood pressure control was not associated with cardiovascular risk [27]. Other observational studies of patients with type 2 diabetes and cardiovascular risk factors have reported associations between lower blood pressure levels and worse outcomes [8–10, 12] but differ from the present analysis in several important aspects. In an observational subgroup analysis of the INVEST trial, which included patients with diabetes and stable coronary artery disease, there was not a statistically significant increase in adjusted mortality rates for patients who achieved and maintained SBP less than 130 mmHg compared with patients who achieved and maintained SBP less than 140 mmHg. However, patients with more than one cardiovascular event were not reported separately [8]. Observational data from two recent trials of inhibitors of dipeptidyl-peptidase 4 have revealed U-shaped relationships between SBP and adjusted CVD rates in patients with type 2 diabetes and established cardiovascular disease or multiple risk factors [9, 10] but did not evaluate the risk of death. In patients with diabetes and high cardiovascular risk who participated in the ONTARGET/TRANSCEND (Ongoing Telmisartan Alone and in Combination with Ramipril Global Endpoint Trial/Telmisartan Randomised Assessment Study in Angiotensin Converting Enzyme Inhibitor Intolerant Subjects with Cardiovascular Disease) trials, on-treatment SBP less than 120 mmHg was associated with higher risk for cardiovascular events and for death, but that study excluded patients with symptomatic heart failure [12]. Another important difference between the present study and those previously cited [8–10, 12, 27] is that we included patients in the early phase after an ACS hospitalization. To the best of our knowledge, only one large study [11] has previously described the relationship between blood pressure and mortality in a similar cohort of patients with type 2 diabetes who recently had been hospitalized with an ACS. In the EXAMINE (Examination of Cardiovascular Outcomes with Alogliptin Versus Standard of Care) trial, baseline SBP less than 120 mmHg or baseline DBP less than 70 mmHg were associated with higher adjusted mortality rates [11]. In ELIXA, we observed similar associations between lower blood pressure and higher mortality only in patients with additional CVD. The reason for this observation remains speculative but may be due to the more extensively adjusted model that was used in the current analysis, which included baseline levels of BNP, a known independent predictor of death with high discriminatory ability [18].

### Study limitations

The most important study limitation is the observational study design, which implies that the described associations do not necessarily represent causal relationships. Although the associations between lower SBP or lower DBP with higher mortality were independent of several known predictors of death, this study did not address potential pathophysiological mechanisms that might explain the relationship between blood pressure and mortality. It is possible that patients with lower blood pressure had other concomitant risk factors that influenced their prognosis. For instance, poor general health, falls and increased arterial stiffness may have contributed but were not measured in the ELIXA trial. Therefore, the present analysis should not be mistaken for a randomized trial of appropriate blood pressure targets, and the results should be interpreted in the context of individualized risk stratification rather than in the context of determining optimal blood pressure treatment targets. Another limitation of the present study was that information regarding coronary anatomy or measurements of coronary artery disease severity were not collected. Inter-arm blood pressure differences are known to be associated with coronary artery disease severity [28] and to predict adverse cardiovascular events in patients with coronary artery disease [29], but were not assessed in ELIXA. Furthermore, LVEF was not available for all patients, and determination of LVEF was made by each study site and not by a core laboratory. Finally, the follow-up period was relatively short, there were few female participants, and the study instructions stated that blood pressure measurements should be performed in the supine rather than in the seated position.

## Conclusions

In this analysis of ELIXA participants with type 2 diabetes and a recent coronary event, a history of additional CVD modified the relationships between SBP or DBP and mortality. Independently of known predictors of death, lower SBP or lower DBP were significantly associated with higher mortality in patients with a history of additional CVD, but not in patients without additional CVD. These findings suggest that patients with a history of multiple cardiovascular events may be more vulnerable to detrimental effects of low blood pressure. When blood pressures measured after an acute coronary event are used to assess the risk of death in patients with type 2 diabetes, the cardiovascular history needs to be taken into consideration.

## Supplementary information

**Additional file 1.** Additional tables and figure.

## Data Availability

The data used to support the findings of this study are available from the corresponding author upon reasonable request.

## Abbreviations

ACCORD: Action to control cardiovascular risk in diabetes
ACS: Acute coronary syndrome
BNP: Brain natriuretic peptide
CHARM: Candesartan in heart failure assessment of reduction in mortality and morbidity
CI: Confidence interval
CVD: Cardiovascular disease
DBP: Diastolic blood pressure
eGFR: Estimated glomerular filtration rate
ELIXA: Evaluation of lixisenatide in acute coronary syndrome
EXAMINE: Examination of cardiovascular outcomes with alogliptin versus standard of care
HIJ-CREATE: The heart institute of Japan candesartan randomized trial for evaluation in coronary artery disease
HOT: Hypertension optimal treatment
HR: Hazard ratio
INVEST: International verapamil SR-trandolapril study
IQR: Interquartile range
ONTARGET: Ongoing telmisartan alone and in combination with ramipril global endpoint trial
PP: Pulse pressure
SBP: Systolic blood pressure
STEMI: ST segment elevation myocardial infarction
T2DM: Type 2 diabetes mellitus
TRANSCEND: Telmisartan randomised assessment study in angiotensin converting enzyme inhibitor intolerant subjects with cardiovascular disease
UKPDS: United Kingdom prospective diabetes study
