# Supplementary material for: Blood pressure and mortality in patients with type 2 diabetes and a recent coronary event in the ELIXA trial
Source: Cardiovasc Diabetol. 2020 Oct 12;19:175. doi: 10.1186/s12933-020-01150-0 (PMC7552471; doi:10.1186/s12933-020-01150-0)
Supplement: Supplementary file 1 — Additional file 1. Additional tables and figure. [file 12933_2020_1150_MOESM1_ESM.docx]

| B-type natriuretic peptide | 130 |
| --- | --- |
| Urinary albumin to creatinine ratio | 69 |
| Blood pressure | 14 |
| Estimated glomerular filtration rate | 13 |
| Heart rate | 12 |
| Diabetes duration | 3 |
| Body mass index | 3 |
| Smoking status | 1 |
| HbA1c | 1 |

**Additional Table S1**. **Number of patients with missing baseline data.**

|  | **Overall (n=4535)**  **HR (95% CI) P**  **per 10 mmHg ↓** | **Index ACS only (n=3527)**  **HR (95% CI) P**  **per 10 mmHg ↓** | **Additional CVD (n=1008)**  **HR (95% CI) P**  **per 10 mmHg ↓** | **P for interaction** |
| --- | --- | --- | --- | --- |
| **Systolic blood pressure** | **1.05 (0.97-1.14) P=0.23** | **0.95 (0.86-1.04) P=0.26** | **1.39 (1.18-1.63) P<0.001** | **<0.001** |
| **Diastolic blood pressure**  **For DBP < 80 mmHg**  **For DBP ≥ 80 mmHg** | *****  **1.44 (1.16-1.78) P=0.001**  **0.83 (0.64-1.07) P=0.16** | **0.94 (0.79-1.12) P=0.48**  **-**  **-** | **1.82 (1.40-2.36) P<0.001**  **-**  **-** | **<0.001** |
| **Pulse pressure** | **1.01 (0.91-1.12) P=0.84** | **0.95 (0.84-1.07) P=0.36** | **1.20 (0.99-1.45) P=0.06** | **0.036** |

**Additional Table S2. Adjusted hazard ratios associated with 10 mmHg lower blood pressure in patients without heart failure. * denotes significantly non-linear associations, where two separate hazard ratios (obtained by piece-wise Cox regression) are reported. Adjustments made for: randomization group, coronary revascularization history, age, sex, self-reported race, heart rate, BMI, smoking status, known duration of diabetes, glycated hemoglobin A1c, use of insulin, eGFR, the logarithm of the urinary albumin to creatinine ratio and the logarithm of the BNP level.**

|  | **Overall (n=3957)**  **HR (95% CI) P**  **per 10 mmHg ↓** | **Index ACS only (n=2352)**  **HR (95% CI) P**  **per 10 mmHg ↓** | **Additional CVD (n=1605)**  **HR (95% CI) P**  **per 10 mmHg ↓** | **P for interaction** |
| --- | --- | --- | --- | --- |
| **Systolic blood pressure** | **1.09 (1.01-1.18) P=0.028** | **0.98 (0.88-1.11) P=0.79** | **1.18 (1.07-1.32) P=0.001** | **0.020** |
| **Diastolic blood pressure** | **1.17 (1.02-1.34) P=0.025** | **0.97 (0.78-1.20) P=0.79** | **1.34 (1.12-1.60) P=0.001** | **0.022** |
| **Pulse pressure** | **1.06 (0.96-1.17) P=0.24** | **0.99 (0.85-1.15) P=0.88** | **1.12 (0.98-1.28) P=0.09** | **0.22** |

**Additional Table S3. Adjusted hazard ratios associated with 10 mmHg lower blood pressure in patients with available information regarding left ventricular ejection fraction (LVEF). Adjustments made for: randomization group, coronary revascularization history, age, sex, self-reported race, heart rate, BMI, smoking status, known duration of diabetes, glycated hemoglobin A1c, use of insulin, eGFR, the logarithm of the urinary albumin to creatinine ratio, the logarithm of the BNP level, and LVEF.**


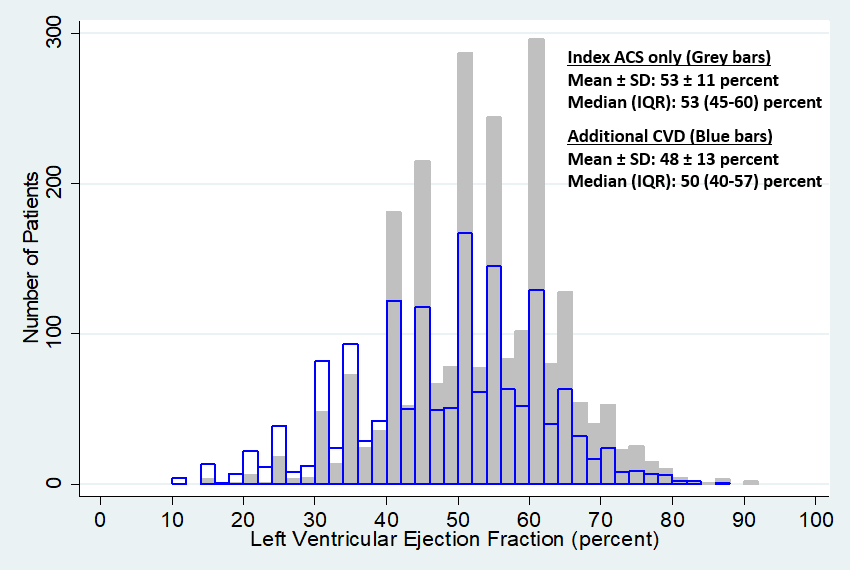


**Additional Figure S1. Baseline distributions of left ventricular ejection fraction. Grey bars show patients with index acute coronary syndrome (ACS) only (n=2352). Blue bars show patients with additional cardiovascular disease (CVD) (n=1605).**
